# LKB1–AMPK Signaling Pathway in Cardiovascular and Other Diseases

**DOI:** 10.1002/mco2.70601

**Published:** 2026-02-24

**Authors:** Zhuo Chen, Qin Yang, Guo‐Wei He

**Affiliations:** ^1^ Department of Cardiovascular Surgery & the Center for Basic Medical Research TEDA International Cardiovascular Hospital, Tianjin University Tianjin China; ^2^ Tianjin Key Laboratory of Molecular Regulation of Cardiovascular Diseases and Translational Medicine Tianjin China; ^3^ The Institute of Cardiovascular Diseases, Tianjin University Tianjin China; ^4^ TEDA International Cardiovascular Hospital, Chinese Academy of Medical Sciences Tianjin China

**Keywords:** AMPK, cardiovascular disease, LKB1, signaling pathway

## Abstract

The LKB1–AMPK signaling pathway is a master regulator of cellular energy homeostasis and a central hub in stress adaptation. As a conserved metabolic sensor, this pathway coordinates glucose, lipid, and protein metabolism, thereby sustaining physiological function across diverse tissues. Beyond its canonical role in energy balance, growing evidence highlights its dysregulation in multiple pathological conditions. Despite extensive mechanistic studies, the disease‐specific regulation and translational potential of the LKB1–AMPK pathway remain incompletely understood. This review systematically studies the molecular basis and regulatory mechanisms of LKB1–AMPK signaling in cardiovascular diseases—including atrial fibrillation, ventricular fibrillation, myocardial infarction, cardiac hypertrophy, heart failure, and atherosclerosis—where impaired pathway activity underlies energy deficits, fibrosis, oxidative stress, and arrhythmogenesis. We further explore its involvement in metabolic disorders such as diabetes and diabetic nephropathy, in neurodegenerative diseases like Alzheimer's and Parkinson's disease, and in oncology, where LKB1 mutations drive tumorigenesis and alter therapeutic responses. Emerging strategies, including metformin, novel AMPK activators, and LKB1‐based gene therapies, are highlighted as promising yet challenged by tissue specificity, off‐target effects, and genetic variation. By integrating insights from cardiovascular, metabolic, neurological, and oncological research, this review underscores the pathway's potential as both a biomarker source and therapeutic target, providing a foundation for precision medicine in complex diseases.

## Graphical abstract

The LKB1–AMPK pathway has a central regulatory role in various diseases. Dysfunction of this pathway can lead to pathological processes in cardiovascular diseases (atrial fibrillation, myocardial infarction, myocardial hypertrophy, atherosclerosis), metabolic diseases (diabetes and kidney disease), neurodegenerative diseases (Alzheimer's disease, Parkinson's disease), and tumors (LKB1 mutation‐driven metabolic reprogramming).

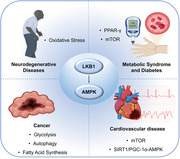

## 1 Introduction

**FIGURE 1:**
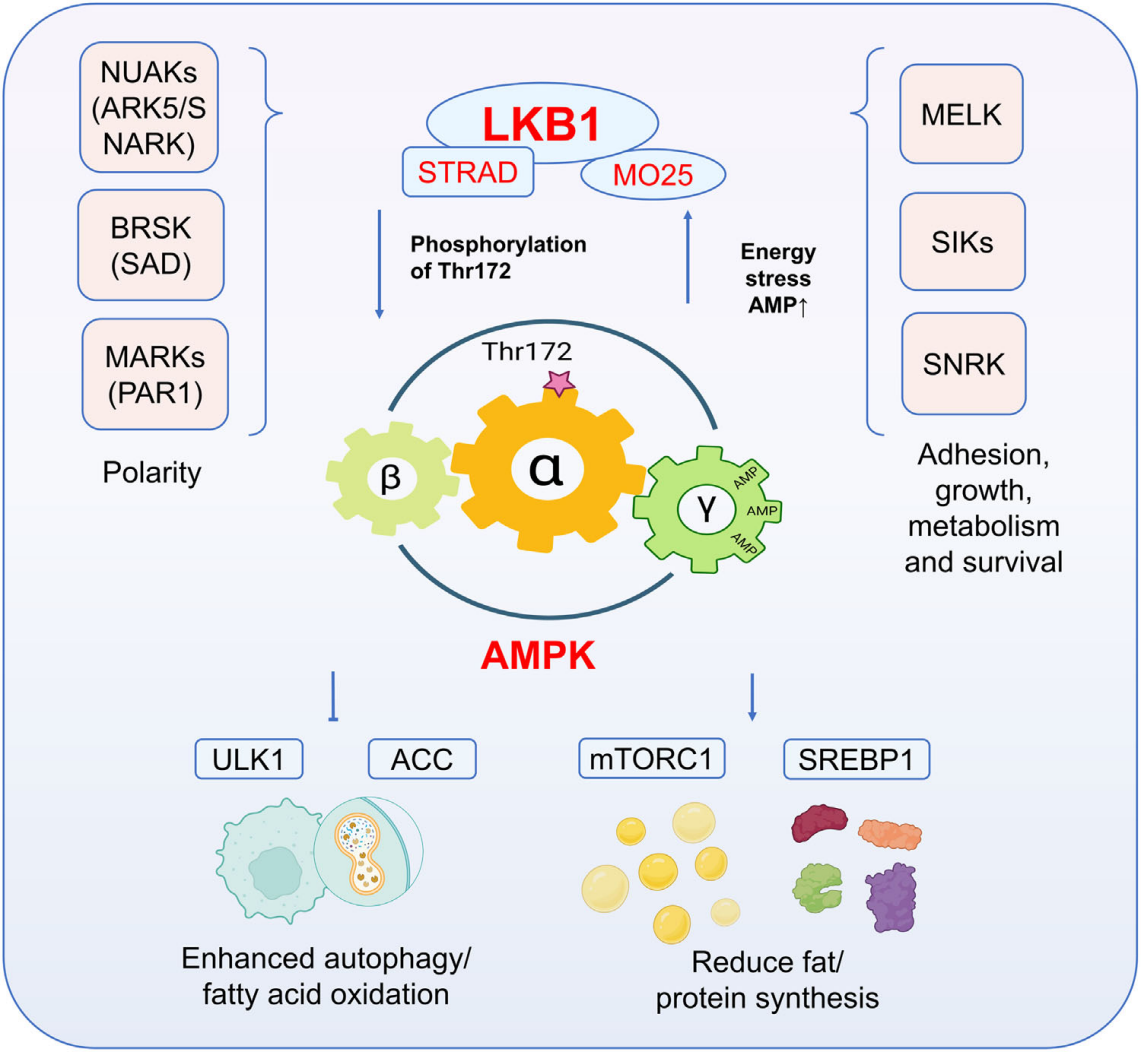
The function of LKB1 and its molecular mechanism in mediating the AMPK pathway. LKB1 forms a stable active complex with STRAD and MO25. Under energy stress conditions, AMP levels increase, primarily binding to the γ regulatory subunit of AMPK, leading to a conformational change in AMPK and promoting its phosphorylation by the LKB1 complex (the key site being Thr172 of the AMPKα subunit). Phosphorylated and activated AMPK then regulates multiple downstream targets: It phosphorylates and activates ULK1 and phosphorylates and inhibits ACC, thereby enhancing autophagy and fatty acid oxidation, respectively; simultaneously, it inhibits mTORC1 activity and the maturation/function of SREBP1, reducing fat and protein synthesis. Additionally, LKB1 phosphorylates and activates a subfamily of AMPK‐related kinases (including NUAKs, BRSKs, MARKs, SNRK, SIKs, MELK, etc.). These kinases regulate key cellular processes such as cell polarity, adhesion, proliferation, metabolism, and survival. The entire LKB1–AMPK signaling cascade collectively coordinates cellular responses to changes in energy status and broadly regulates various physiological functions. Some elements in this figure were created using BioRender.

The liver kinase B1 (LKB1)‐AMP‐activated protein kinase (AMPK) pathway is a master regulator of cellular energy homeostasis and a conserved mechanism that enables cells to adapt to metabolic stress [1, 2]. AMPK functions as an intracellular energy sensor, activated by an increased AMP/ATP ratio, upstream kinases including LKB1, CaMKKβ, and TAK1, and various stress signals [3, 4, 5]. Once activated, AMPK orchestrates diverse downstream processes such as glucose uptake, fatty acid oxidation, autophagy, mitochondrial biogenesis, and inhibition of protein synthesis through suppression of the mechanistic target of rapamycin (mTOR) pathway [6, 7, 8]. These multifaceted roles place AMPK at the intersection of metabolism, stress response, and disease progression (see Figure 1).

Beyond its canonical role in metabolic regulation, dysregulation of the LKB1–AMPK pathway has been implicated in cardiovascular, metabolic, neurological, and oncological disorders [9, 10, 11, 12]. In cardiovascular disease, impaired AMPK activity exacerbates ischemia/reperfusion injury, promotes maladaptive cardiac hypertrophy, and contributes to atrial fibrillation (AF) and heart failure (HF) [13, 14, 15, 16]. In metabolic disorders such as obesity, insulin resistance, diabetes, and non‐alcoholic fatty liver disease (NAFLD), AMPK dysfunction leads to lipid accumulation, oxidative stress, and impaired glucose handling [17, 18, 19, 20]. Similarly, in neurodegenerative diseases, including Alzheimer's and Parkinson's disease, AMPK dysregulation influences autophagy, synaptic plasticity, and neuronal survival [21, 22, 23]. In oncology, mutations in the upstream kinase LKB1 (STK11) are frequent in lung adenocarcinoma and other cancers, driving tumor progression and therapeutic resistance [24, 25, 26].

Despite extensive mechanistic research, significant gaps remain in understanding disease‐specific regulation of the LKB1–AMPK pathway. Existing literature often focuses on single organ systems or isolated mechanisms, whereas a comprehensive cross‐disease synthesis is still lacking. Furthermore, translation of preclinical insights into clinical interventions has been limited by challenges such as tissue‐specific responses, off‐target effects, and patient genetic variation, all of which are underscored by recent experiences with direct AMPK activators in animals and humans [27, 28].

This review, therefore, aims to provide a systematic and integrative overview of the LKB1–AMPK pathway across multiple disease contexts. First, we summarize the fundamental biology of LKB1–AMPK signaling and its upstream regulators. Next, we analyze its diverse roles in cardiovascular disease, metabolic disorders, neurodegenerative disease, and cancer, highlighting mechanistic commonalities and disease‐specific features. We then focus on therapeutic targeting of the LKB1–AMPK pathway, where we review current pharmacological activators (e.g., metformin, salicylate, AICAR), novel direct activators (PF‐06409577, MK‐8722, PXL770), and their therapeutic potential alongside major translational challenges [28]. Finally, we discuss future perspectives, emphasizing the need for isoform‐ or tissue‐selective modulators, targeted delivery strategies, and biomarker‐guided precision medicine approaches.

By integrating insights from basic research and clinical studies, this review underscores the LKB1–AMPK pathway's role as both a biomarker source and therapeutic target. The comprehensive analysis provided here not only highlights mechanistic insights but also positions the pathway as a transformative axis for precision therapy across cardiovascular, metabolic, neurodegenerative, and oncological diseases.

## 2 Molecular Architecture and Physiological Functions of the LKB1–AMPK Pathway

Based on the established role of the LKB1–AMPK pathway as a core regulator of cellular energy homeostasis and a pivotal hub for stress adaptation, a thorough analysis of its molecular structural characteristics and physiological functional networks is essential for understanding the pathway's function in maintaining health and driving disease development.

### 2.1 Regulation of Cellular Energy Metabolism

The LKB1–AMPK pathway does not operate as a single on/off “fuel gauge.” It is spatially and compositionally diversified into microdomains that tune substrate choice and stress tolerance. Live‐cell reporters reveal that AMPK activity rises with distinct kinetics at lysosomes versus mitochondria, and even shuttles to the nucleus, implying location‐specific substrate triage under stress rather than a uniform global switch [29]. At the enzyme level, β‐subunit allostery is not merely modulatory noise; phosphorylation of AMPKβ1‐Ser108 is required to sustain fatty‐acid oxidation (FAO), mitochondrial biogenesis, and lipophagy during lipid load, thereby coupling nutrient context to AMPK efficacy [30]. This mechanistic layer dovetails with the emerging pharmacology of ADaM‐site ligands. Naturally occurring dihydrophenanthrenes directly engage the β‐subunit ADaM pocket, stabilize activation, and depress de novo lipogenesis in primary hepatocytes. This provides proof that small‐molecule tuning of β‐allostery can re‐weight cardiac substrate usage without massive ATP depletion [31, 32].

Cardiac and atrial phenotypes underscore the translational stakes. Atrium‐selective AMPK deletion provokes early electrical remodeling, connexin/ion‐channel reprogramming, and spontaneous AF, arguing that baseline AMPK tone is a constitutive guardian of atrial metabolic–electrical homeostasis, not just emergency braking [14]. Complementarily, unbiased profiling in human atrial tissue links AMPK to preservation of oxidative metabolism and contractile integrity, while genetic/functional perturbation reprograms substrate selection and primes arrhythmogenesis [16]. At the whole‐cell scale, AMPK's control of FAO is context dependent. Classical ACC2 control of malonyl‐CoA remains relevant, but cardiac FAO and function can be maintained via compensatory routes when AMPK–ACC signaling is disrupted, cautioning against simplistic ACC‐centric models in the heart [33, 34, 35]. Taken together, a modern view is that (i) compartmentalized AMPK pools, (ii) β‐subunit allostery/phosphorylation, and (iii) tissue‐specific redundancies collectively shape myocardial energy routing. This architecture invites precision therapies (e.g., isoform or ADaM‐biased activators) rather than global AMPK agonism [30, 31, 32, 33, 34, 35].

### 2.2 Control of Cell Growth and Autophagy

The long‐standing paradigm in this field, namely that energy stress activates AMPK, which in turn activates ULK1, culminating in autophagy induction, is being revised. Recent evidence shows AMPK can restrain ULK1 and dampen acute autophagy induction during energy crisis, while paradoxically preserving the autophagy machinery from caspase degradation to enable delayed recovery once stress abates [36, 37, 38]. This dualism explains why forced AMPK upshifts sometimes blunt early autophagic flux, yet improve survival after stress release. It also aligns with new phosphosite‐centric maps revealing reciprocal AMPK–mTOR circuitry that is more bidirectional and substrate‐specific than previously appreciated. mTORC1‐dependent phosphorylation of AMPK subunits and AMPK phosphorylation of mTORC1 gatekeepers (e.g., raptor/TSC2) create state‐dependent “who controls whom” outcomes [39, 40]. AMPK's suppression of mTORC1 can also proceed via nutrient‐sensing modules such as GATOR2/WDR24, linking glucose status to Rag–lysosome signaling independently of bulk ATP [39].

Mitophagy control likewise appears split: AMPK activation can curb mitophagy of functional mitochondria, yet enhance clearance of damaged ones, a substrate‐quality filter that reconciles prior contradictory cardioprotection reports [41]. In the cardiac conduction system, AMPK intersects with proteostatic quality control of ion channels; during ischemia–reperfusion, AMPK phosphorylates Nav1.5 to expose an LC3‐interacting region, triggering selective autophagic degradation, which is an adaptive but potentially pro‐arrhythmic tradeoff if overactivated [42]. Conceptually, these datasets support the “adaptive gating” model. AMPK prioritizes ATP salvage by curbing growth programs (mTORC1), delays bulk autophagy to avoid catastrophic biomass loss, but maintains scaffold integrity (ULK1 complex, mitophagy for damaged organelles) to enable staged recovery [36, 37, 39, 40, 41, 42, 43]. Therapeutically, this favors time‐ and compartment‐resolved AMPK modulation (e.g., β1‐biased activators that spare ULK1‐inhibitory phosphosites) rather than chronic systemic activation.

### 2.3 Maintenance of Vascular Homeostasis

Vascular protection by AMPK is not only metabolic, it is mechano‐metabolic. Under atheroprotective flow, endothelial KLF4 induction couples to AMPK to suppress PFKFB‐driven glycolysis, reallocating ATP for junctional remodeling and anti‐inflammatory programs—an elegant explanation for how low‐glycolytic endothelium maintains quiescence despite shear work [44]. Direct force on VE‐cadherin recruits LKB1 to activate AMPK, which boosts eNOS activity and glucose uptake, and reinforces the cadherin–actin complex; blocking this axis abrogates flow‐aligned cytoskeletal architecture and vasodilatory competence [45]. This positions AMPK as a junction‐proximal “power supply,” matching local ATP production to barrier strengthening and NO bioavailability. Notably, AMPK effects are context‐dependent across vascular beds: acute AMPK–eNOS signaling can increase permeability in the retina, while chronic mechano‐activation stabilizes arterial junctions—differences likely rooted in local scaffolds, shear waveforms, and eNOS/Src co‐signaling [46]. Small‐molecule or natural‐product AMPK activators that improve endothelial function have entered preclinical vascular models, but isoform bias and vascular‐bed heterogeneity remain under‐addressed variables [31, 47].

Overall, the LKB1–AMPK pathway maintains vascular homeostasis by (i) coupling adhesion mechanotransduction to metabolic upshifts at junctions; (ii) re‐programming glycolysis via KLF4 to a quiescent, anti‐inflammatory state; and (iii) calibrating NO and barrier integrity in a bed‐ and stimulus‐specific manner [44, 45, 46, 47]. A forward‐looking thesis emerges: precision AMPK therapy should be spatial (microdomain‐targeted), temporal (stress‐phase aware), and isoform‐biased, especially in atrial disease where energy–adhesion–electrophysiology forms a vulnerable triad.

## 3 The LKB1–AMPK Pathway as a Central Player in Cardiovascular Diseases

Energy supply and stress adaptation underpin cardiovascular homeostasis. Serving as both a metabolic sensor and a coordinator of stress responses, the LKB1–AMPK pathway couples substrate use to protective signaling in cardiomyocytes and vascular endothelium, shaping ion‐channel expression and coupling, mitochondrial quality control, inflammasome restraint, endothelial nitric oxide production, and macrophage cholesterol handling [14, 48, 49, 50, 51]. In the process of disease progression, pathway activity has undergone stage‐ and cell‐specific reprogramming, which makes it impossible to be optimal to activate or inhibit it comprehensively and continuously. Therapeutic reasoning, therefore, favors interventions that tune dose, timing, and tissue context to the disease‐course window and microenvironment [14, 48, 49, 52, 53].

### 3.1 Cardiac Arrhythmias

**FIGURE 2:**
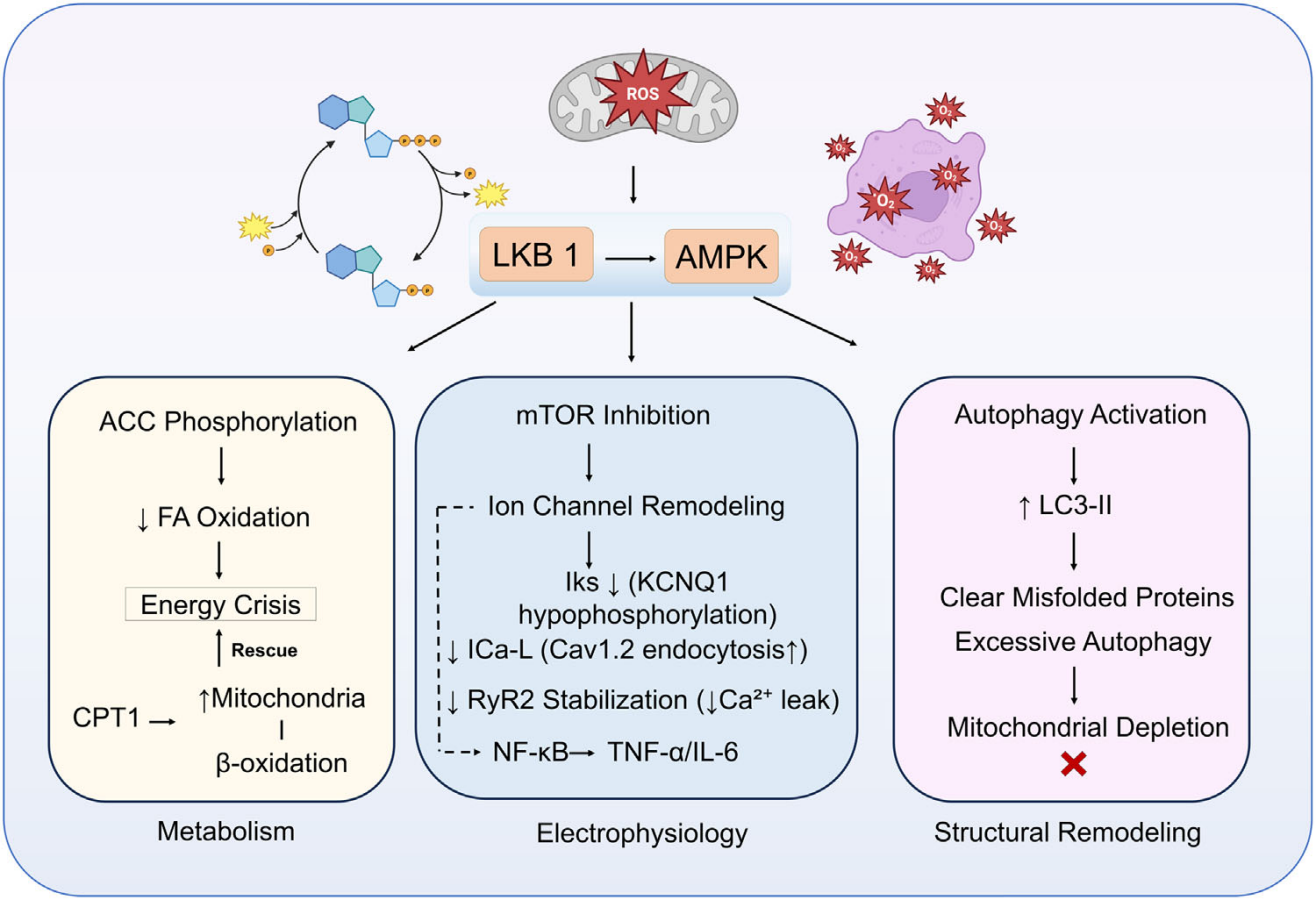
Multidimensional roles of LKB1/AMPK signaling in atrial fibrillation. Energy stress (ROS/AMP↑) activates AMPK via LKB1‐mediated phosphorylation, driving metabolic imbalance (ACC/CPT1‐regulated fatty acid oxidation dysregulation), electrophysiological remodeling (mTOR inhibition‐induced IKs/ICa‐L/RyR2 dysfunction), and dual autophagy effects (protective clearance ↔ excessive mitochondrial depletion), collectively forming the AF substrate. Some elements in this figure were created using BioRender.

In the conditioned mouse models, the dual deletion of atrial‐specific AMPKα1/α2 can lead to the delay of atrial conduction and repolarization duration and the increase in abnormal triggered activity, prior to the onset of significant structural changes. This is accompanied by atrial‐specific downregulation of the transcription of gap junction proteins and multiple ion channel subunits. These electro‐molecular changes precede the subsequent development of left atrial interstitial fibrosis, suggesting that AMPK exerts a fundamental protective effect on atrial electrical homeostasis [14]. Independent studies further used stable isotope tracing and LC‐MS/MS quantification to show that atrial AMPK deletion shifts the substrate preference of atrial mitochondria from fatty acid oxidation to pyruvate oxidation. It also extensively downregulates proteins related to fatty acid uptake and oxidation, while simultaneously reducing the expression of the PGC1‐α/PPARα pathway. Metabolic reprogramming and electrophysiological abnormalities together constitute a pro‐fibrillatory substrate [48]. Advanced glycation end products (AGEs) can reduce the expression of Cx43/Cx40 by inhibiting AMPK signaling, disrupting the topological stability of gap junctions. This provides another evidence chain for metabolism–structure coupling [49]. In addition, the metabolic intermediate succinic acid is elevated in obesity‐ and diabetes‐related AF‐susceptible models. Exogenous succinic acid loading can induce mitochondrial damage, gap junction lateralization, and fibrosis through its receptor SUCNR1 and AMPK dephosphorylation, thereby increasing AF susceptibility. Activation of AMPK by AICAR can reverse this phenotype [50]. In diabetes‐related AF, low activity of the atrial FNDC5–AMPK pathway is associated with impaired mitochondrial dynamics and activation of the NLRP3 inflammasome. This suggests that the metabolism–inflammation crosstalk is a key intervenable target [51]. The sequential relationship of “metabolic stress triggering—AMPK activity change—electrophysiological and structural remodeling” shown in Figure 2 is consistent with the aforementioned animal and cellular evidence. Among them, gap junctions, ion channel expression regulation, and mitochondrial quality control constitute three major downstream pathways. These pathways appear prior to overt fibrosis and conduction dispersion, highlighting the potential value of early metabolic intervention [14, 48, 49, 50, 51].

Several lines of evidence support an intervention strategy based on disease stages. In the early metabolic‐electrophysiological stage, priority should be given to stabilizing atrial electrical synchronization by improving fatty acid–glucose substrate utilization and maintaining gap junction protein expression. In animal experiments, AMPK agonists or enhanced upstream mechanical–metabolic coupling are associated with the stabilization of electrical activity [14, 48, 49, 52, 53]. In the structural remodeling stage, combined regimens targeting inflammasomes and fibroblast metabolism are more logical. This is because they can alleviate tissue stiffening and electrical heterogeneity without increasing triggering activity [51, 54, 55]. In clinical scenarios related to pacemakers, the site of electrical stimulation and conduction synchronization also interacts with metabolic pathways. Physiological pacing in the Bachmann's bundle, His bundle, or left bundle branch regions can shorten the overall atrial activation time and improve mechano‐electrical coupling. This is expected to reduce the incidence of pacing‐related AF, suggesting that systematic optimization of mechanics–metabolism–electrophysiology may bring additive benefits at the patient level [53].

Based on the above evidence, interventions targeting the LKB1–AMPK pathway for AF can be advanced at two levels. First, establishing dosage and timing selections based on the “metabolism–electrophysiology–structure” three‐stage window can avoid increased susceptibility of ectopic pacemakers caused by simple enhancement of metabolic flux in the late stage of severe fibrosis [14, 48, 49, 50, 51, 54]. Second, a multi‐target strategy combining upstream mechanical force sensing and endothelial–myocardial coupling signals—such as maintaining endothelial–metabolic homeostasis while stabilizing the atrial gap junction network—can reduce the sources of electrical heterogeneity [52, 53]. Theoretically, selective agonists that directly activate specific subunits or target restricted cell types are more conducive to reducing off‐target effects. However, systematic evaluation of the exposure–effect relationship and safety margins of pharmacokinetics in atrial tissue is required at the clinical trial level [52, 56, 57].

**FIGURE 3:**
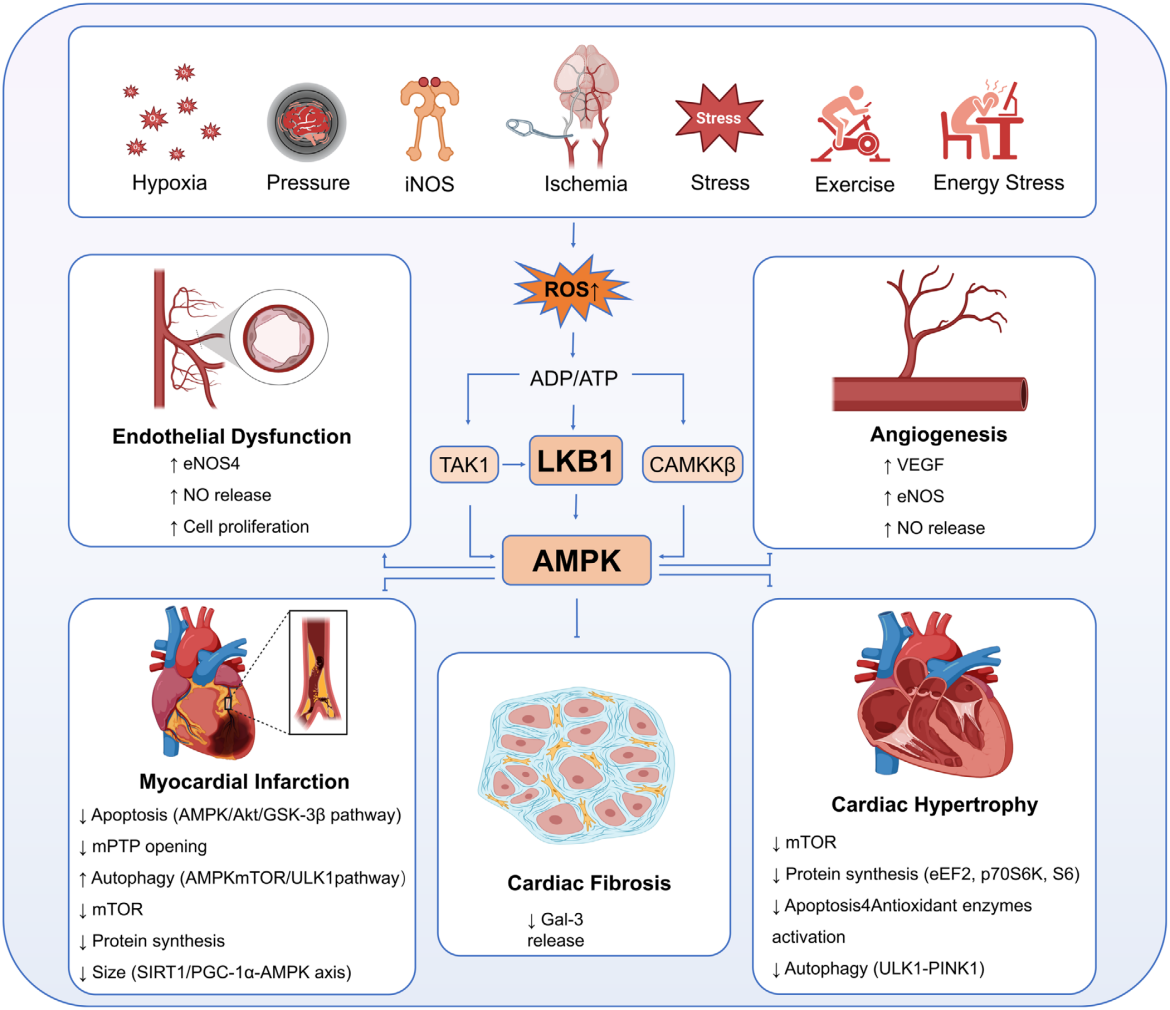
The regulatory role of AMPK in various physiological and pathological processes in the cardiovascular system. Various stimuli (such as hypoxia, ischemia, stress, oxidation, overload, stress, iNOS, exercise, energy stress, etc.) induce the production of ROS (reactive oxygen species), which affect ADP/ATP, activate upstream pathways such as TAK1, LKB1, and CaMKKβ, and thereby regulate AMPK. AMPK is involved in angiogenesis (upregulating VEGF, eNOS, and promoting NO release), cardiac hypertrophy (regulating mTOR, protein synthesis, apoptosis, antioxidant enzyme activation, and autophagy), endothelial dysfunction (by affecting eNOS4, NO release, and cell proliferation), myocardial infarction (involving apoptosis, mPTP opening, autophagy, mTOR, protein synthesis, size regulation, and other pathways), as well as Gal‐3 release and autophagy‐related processes. This highlights AMPK's central regulatory role in the cardiovascular pathophysiological network. Some elements in this figure were created using BioRender.

In the ventricular level, arrhythmias induced by small‐molecule targeted drugs provide a natural experiment for observing the role of AMPK in ventricular electrical homeostasis. Bruton tyrosine kinase inhibitor ibrutinib can induce abnormal action potential repolarization, sarcoplasmic reticulum calcium leakage, and decreased conduction velocity. These effects are accompanied by increased susceptibility to ventricular fibrillation. In rats and isolated hearts, decreased phosphorylation of myocardial AMPK protein is observed. Short‐term administration of AICAR can partially correct abnormalities in action potentials and calcium transients, and reduce inducibility [58]. Independent cohorts and reviews have also linked ibrutinib‐related ventricular events to calcium handling disorders, mitochondrial stress, and proinflammatory signals. This suggests that the metabolism–calcium homeostasis–gap junction triangular network is also important at the ventricular level [59, 60, 61]. Figure 3 presents the coupling points of ventricular arrhythmogenesis through the triangular relationship of “AMPK–calcium cycling–gap junctions.” It also labels several types of drugs and signals intersecting with this network on the edges, facilitating the identification of risk nodes for drug–biological interactions at the pathway level [58, 59, 60, 61].

In the risk management of ventricular fibrillation or generalized ventricular arrhythmias, preventive strategies targeting drug‐induced metabolic‐calcium handling damage are feasible. Specifically, in patients requiring relevant targeted drugs, prior assessment of previous myocardial metabolic vulnerability and electrophysiological baseline risk should be prioritized. This should be combined with reversible AMPK pathway modulators for short‐term protective intervention during the peri‐therapeutic period. Animal studies have provided relevant clues [58, 59, 60, 61]. Using metabolism–conduction coupling as a comprehensive indicator, combined with non‐invasive or minimally invasive phenotypic assessment (electrical axis dispersion, conduction heterogeneity, and myocardial stress markers), intervention can be implemented in the early stage when coupling disruption is indicated. This avoids progression to the late refractory stage dominated by scar tissue and heterogeneity [58, 59, 60, 61]. The pharmacokinetics of direct systemic AMPK activators in the myocardium, the risk of myocardial glycogen accumulation, and the safety of combination with existing anti‐arrhythmic drugs still need to be clarified through a stratified design and exposure‐response analysis in clinical practice. Empirical extrapolation should be avoided.

### 3.2 Myocardial Infarction (MI) and Ischemic Preconditioning

During acute ischemia‐reperfusion, rapid activation of AMPK can inhibit mTORC1 and promote ULK1‐mediated autophagy‐mitophagy. It also regulates fatty acid oxidation and glucose uptake to maintain the balance between energy supply and demand. Multiple animal studies have observed an association between increased activity of the AMPK–ULK1 pathway and reduced ischemia‐reperfusion injury under different compounds or genetic backgrounds [41, 62, 63, 64]. At the same time, AMPK alleviates the post‐reperfusion inflammatory cascade by inhibiting the activity of the NLRP3 inflammasome and related inflammatory pathways. This indirectly reduces cardiomyocyte pyroptosis and interstitial activation [54, 65, 66, 67]. Notably, recent cellular and tissue studies suggest that AMPK does not simply promote “the more, the better” for mitophagy. Instead, it tends to promote the preferential clearance of damaged mitochondria and limit excessive phagocytosis of functional mitochondria. This “quality selectivity” explains the observed differences in total autophagy levels across different models [41, 68, 69].

**FIGURE 4:**
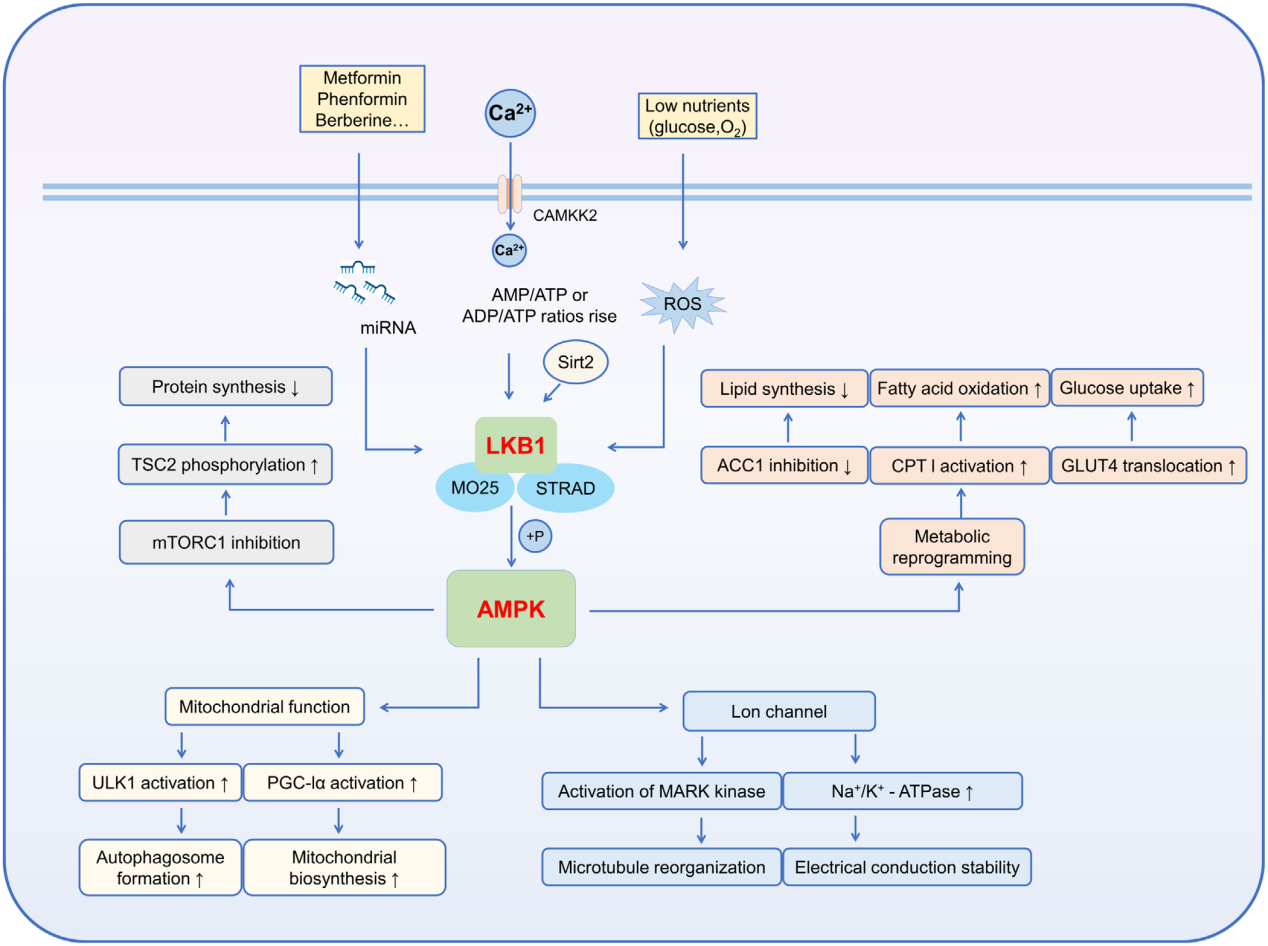
LKB1–AMPK pathway cross‐disease regulatory network and therapeutic intervention modules. Energy stress (nutritional deficiency/hypoxia) or therapeutic drugs (metformin/berberine, etc.) trigger AMPK phosphorylation activation by increasing the AMP/ATP ratio, activating CAMKK2, or the LKB1–STRAD–MO25 complex (with Sirt2 co‐activation), thereby inhibiting ACC1 to reduce lipid synthesis, activating CPT1/GLUT4 to promote fatty acid oxidation and glucose uptake, and achieving metabolic reprogramming. Simultaneously activating ULK1 to enhance autophagy and clear damaged components, upregulating PGC‐1α to promote mitochondrial biogenesis and improve energy supply, and regulating Na^+^/K^+^‐ATPase activity and microtubule reorganization to maintain ion channel homeostasis. Ultimately, this process coordinates multisystem disease treatment by inhibiting the mTORC1 pathway through TSC2 phosphorylation.

Figure 4 divides the time course of acute MI‐reperfusion into three stages. The early ischemic stage is centered on an energy crisis and an ion imbalance. The role of AMPK mainly manifests in maintaining metabolic order and initiating acute autophagy. In the early reperfusion stage, oxidative stress and inflammasome activation become the main drivers. AMPK exerts protective effects by limiting NLRP3 activity, maintaining mitochondrial membrane potential, and promoting the selective clearance of damaged mitochondria. In the subacute stage, the metabolic‐inflammatory crosstalk between cardiomyocytes, fibroblasts, and immune cells determines scar formation and left ventricular remodeling. AMPK has potential value in regulating macrophage cholesterol metabolism and polarization, as well as fibroblast glycolysis and differentiation [41, 52, 54, 62, 63, 64, 65, 66, 67, 69]. This figure emphasizes the stage‐specificity and cell lineage specificity rather than unidirectional changes in a single indicator.

Based on stage‐specific characteristics, interventions for MI and reperfusion therapy can be carried out in three time windows. The first time window—from immediate reperfusion to several hours later—focuses on short‐term AMPK upregulation, combined with mitochondrial protection to achieve “preferential clearance” and energy re‐homeostasis. This avoids “accidental damage” to recoverable mitochondrial populations due to excessive autophagy [41, 68, 69]. The subacute stage during hospitalization serves as the second time window. Combined interventions targeting the inflammasome–metabolism crosstalk are implemented here. Recent animal studies support the inhibition of pyroptosis and improvement of cardiac aging phenotype and left ventricular function through the AMPK–NLRP3 pathway. This provides a basis for the “metabolism–inflammation integration” regimen [66, 67]. The third time window—early remodeling stage—focuses on the interactions between cardiomyocytes, immune cells, and fibroblasts. Selective activation of the AMPKβ1 subunit in macrophages can downregulate cholesterol synthesis and inflammatory genes, and enhance the activity of autophagy‐related pathways. In atherosclerotic models, this has been shown to reduce inflammatory burden and lesion volume. This mechanism has translational potential in regulating the inflammatory microenvironment after MI. However, verification of myocardial exposure and effects in the MI context is required [45]. Regarding pharmacological challenges, direct macromolecular or small‐molecule agonists need to balance exposure differences between myocardial and immune cells and metabolic side effects in peripheral tissues. Systemic strong activation may lead to myocardial glycogen deposition or unintended interactions with other metabolic pathways. Molecular design and delivery strategies should tend toward selectivity [45, 56].

### 3.3 Pathological Cardiac Hypertrophy and Heart Failure

**FIGURE 5:**
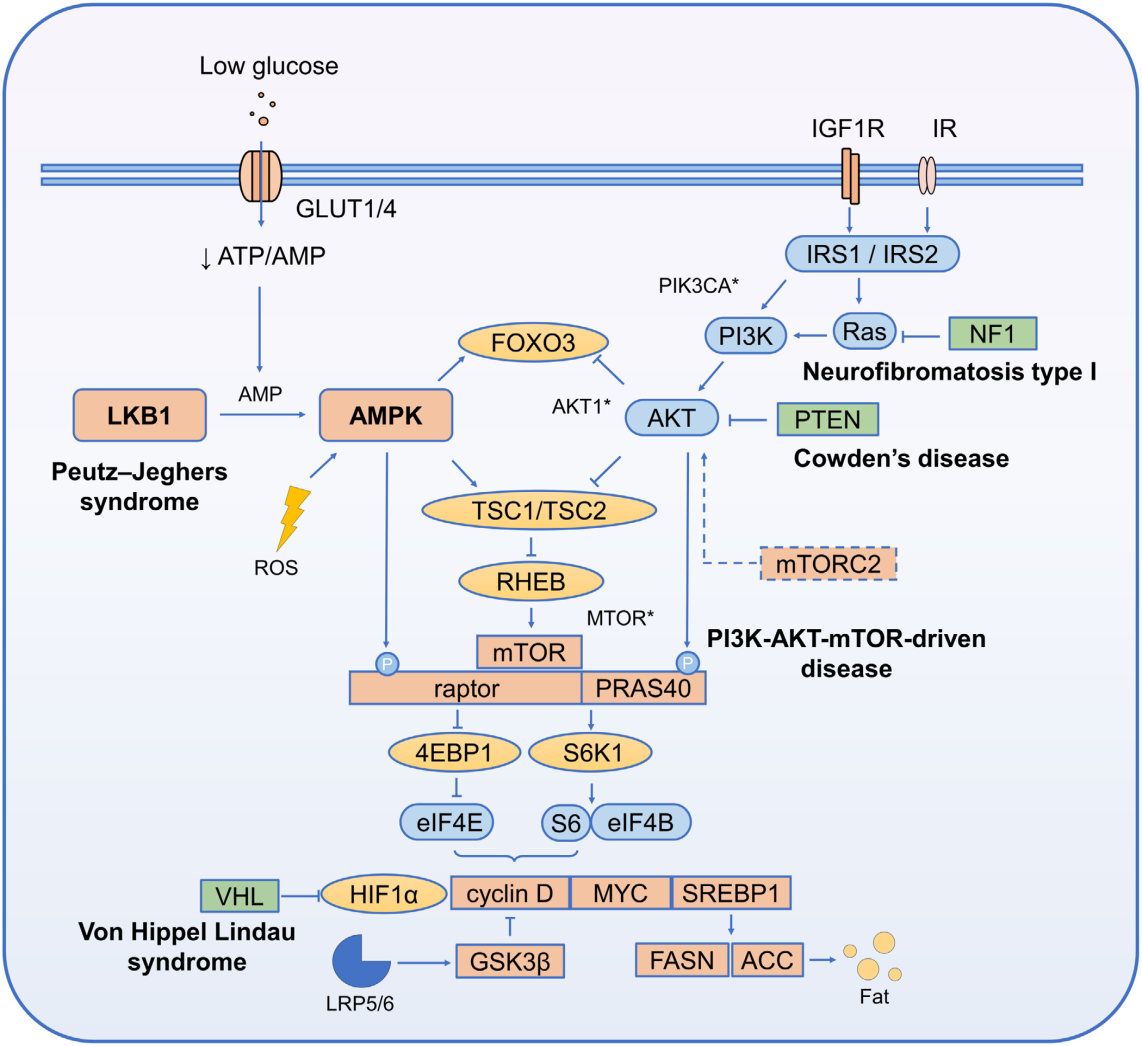
The AMPK and PI3K pathways converge and antagonistically regulate multiple downstream effectors, including the mTOR complex. This schematic diagram illustrates the mechanism by which AMP‐activated protein kinase (AMPK) and phosphoinositide 3‐kinase (PI3K) pathways converge and antagonistically regulate multiple downstream effectors, including the mTOR complex. Low glucose levels enter the cell via glucose transporters (GLUT1/4), leading to a change in the ATP/AMP ratio and activating AMPK mediated by liver kinase B1 (LKB1). AMPK negatively regulates the mTOR complex by inhibiting the tuberous sclerosis complex (TSC1/TSC2) and other mechanisms. Concurrently, activation of the insulin receptor (IR) and insulin‐like growth factor 1 receptor (IGF1R) activates PI3K via insulin receptor substrate (IRS1/IRS2), which further activates protein kinase B (AKT). The mTOR complex 2 (mTORC2) participates in the phosphorylation regulation of AKT. Following AKT activation, TSC1/TSC2 inhibition is relieved, thereby activating the mTOR complex. The two pathways converge at the mTOR complex, exerting opposing regulatory effects on downstream effectors such as eukaryotic initiation factor 4E‐binding protein 1 (4EBP1) and ribosomal S6 kinase 1 (S6K1), thereby exerting different influences on cellular processes such as proliferation and metabolism. Additionally, the figure highlights various disease‐associated gene mutation sites (e.g., PIK3CA*, AKT1*, MTOR*) and related diseases, such as Peutz–Jeghers syndrome, Cowden disease, neurofibromatosis type 1, and von Hippel–Lindau syndrome, underscoring the critical role of this pathway in disease onset and progression.

Metabolic reprogramming is the underlying driver of the progression from cardiac hypertrophy to decompensation. Multi‐omics studies suggest that in the early stage of hypertrophy, glycolysis is enhanced and fatty acid oxidation is inhibited. As the load persists, the quantity and quality of the mitochondrial network decrease simultaneously. The tension between protein synthesis and organelle renewal continues to increase. AMPK acts as a “distribution valve” for this tension. Its inhibition of ribosome biogenesis and translation elongation, as well as its promotion of autophagy and mitochondrial biogenesis, determines whether the myocardium can maintain structural stability under energy deficiency. As suggested in Figure 5, regarding the interaction with mTORC1, recent studies have identified the hierarchical balance between Raptor and the TSC complex as key regulatory nodes. Phosphorylation events at these nodes “downshift” the protein synthesis pathway under long‐term stress. This creates space for autophagy and mitochondrial quality control [6, 70]. AMPK prioritizes “slowing down” synthesis in the early stage of stress. It then gradually releases the inhibition after energy status stabilizes to avoid sarcomeric protein loss and contractile function decline caused by long‐term strong inhibition.

At the structural and ultra‐structural levels, recent cellular studies suggest that microtubule network proliferation and disorganized arrangement in hypertrophic cardiomyocytes can increase cytoplasmic viscosity and affect calcium cycling. AMPK activation reduces the excessive accumulation of microtubules by “reducing the burden” of protein synthesis and folding, and by upregulating the autophagy pathway. This improves the geometric relationship between myofilaments and T‐tubules, providing conditions for the homogenization of calcium transients [71]. Correspondingly, glucose transport at the myocardial energy input end is also regulated by AMPK. The classic TBC1D1–GLUT4 translocation pathway remains effective. However, the more critical issue is how to avoid excessive glycogen deposition under long‐term stress. Experience from insulin‐independent activation strategies shows that sustained strong activation can induce increased glycogen load and myocardial weight in both skeletal muscle and the heart. This suggests that “whole‐system, whole‐time” activation is not advisable. Tissue‐specific and stage‐specific precise activation is more in line with the needs of long‐term HF management [27, 72, 73, 74].

Regarding autophagy and mitochondrial homeostasis, recent experiments and reviews consistently indicate that AMPK promotes the clearance of damaged mitochondria and mitochondrial biogenesis through two mechanisms. On one hand, it inhibits mTORC1. On the other hand, it regulates initiation complexes such as ULK1. For animal models of the transition from hypertrophy to HF, the speed and magnitude of this “turnover” directly determine the metabolic recovery window of the myocardium. If inflammasome activation or redox imbalance is also present, the anti‐inflammatory and antioxidant effects of AMPK can lower the activation threshold of NLRP3. This indirectly reduces cardiomyocyte pyroptosis and the transmission of fibrotic signals, thereby delaying the decline in left ventricular compliance and the deterioration of ejection fraction [75, 76, 77, 78].

Repeatable details related to AMPK have also emerged in the field of electrophysiology. Cardiomyocytes in HF often exhibit increased intracellular sodium load and impaired intracellular calcium extrusion. In the last 2 years, translational studies have attributed part of this mechanism to the upregulation of sodium–hydrogen exchanger activity and prolonged late sodium current‐induced depolarization pressure. Although AMPK is not a direct switch for these channels, it can downregulate the driving force of multiple pro‐depolarization pathways by regulating cellular energy status and stress kinases. This forms an indirect inhibition effect characterized by “energy priority and membrane stabilization first.” In parallel, the anti‐arrhythmic and anti‐remodeling effects of SGLT2 inhibitors in HF have gradually become clear. Their sustained inhibition of sodium–hydrogen exchangers at the ion homeostasis level echoes the positive regulation of AMPK signaling at the metabolic level. This “metabolism plus ion” dual correction pathway deserves further bridging studies between molecular mechanisms and clinical endpoints [79, 80, 81, 82, 83].

In the early compensatory stage of hypertrophy, the focus should be on inhibiting excessive protein synthesis and maintaining mitochondrial quality. In the decompensatory stage or when inflammation is amplified, the focus shifts to inhibiting NLRP3 and reconstructing the metabolic network. Systemic, long‐term activation strategies have shown risks of increased myocardial glycogen load and myocardial weight. Future pharmacokinetic designs centered on β‐subunit selectivity or myocardial targeting will be safer [27, 72, 73, 74, 84, 85]. Potential “antagonistic or synergistic” boundaries exist in the administration sequence and dosage of SGLT2 inhibitors, mTORC1 inhibitors, and AMPK modulators. Clinical protocols need to be dynamically calibrated based on inflammatory markers, metabolic imaging, and myocardial energy spectroscopy [79, 80, 81, 82, 83, 86].

### 3.4 Atherosclerosis and Vascular Dysfunction

The sensing of hemodynamic mechanical forces by endothelial cells and metabolic reprogramming determines the regional susceptibility to plaque formation. Mechanical forces acting on VE‐cadherin in endothelial adherens junctions can activate LKB1 upstream, which in turn activates AMPK. This forms a coupling chain from “mechanics to metabolism to signaling.” The establishment of this chain endows endothelial cells with metabolic flexibility under fluctuating shear stress. This helps maintain barrier function, endothelial nitric oxide synthase activation, and an anti‐inflammatory phenotype [45]. The study on glucose metabolism profiles is also advancing. Rate‐limiting nodes of endothelial glycolysis, such as PFKFB3, are upregulated under unsteady shear stress and inflammatory microenvironments. The “ratio adjustment” of glycolysis and mitochondrial oxidation by AMPK determines whether endothelial cells move toward defensive anti‐inflammation or slide into the pro‐disease trajectory of neovascularization and endothelial‐to‐mesenchymal transition (EndMT). Inhibiting excessive PFKFB3 activation under moderate AMPK activation can partially restore endothelial metabolic homeostasis. This reduces endothelial–mesenchymal transition and pathological neovascularization, thereby improving plaque vulnerability characteristics [52, 87, 88, 89].

Single‐cell resolution studies have provided new clues to the fate transition of macrophages and smooth muscle cells. Integrated single‐cell atlases of human carotid plaques show that smooth muscle cells exhibit lineage drift toward osteoblast‐like and macrophage‐like phenotypes. Macrophages, on the other hand, form a multi‐subpopulation division of labor among inflammation, lipid handling, and foam cell formation. In the integrated analysis of multi‐datasets from mice and humans, the proportion and function of these subpopulations match the characteristics of plaque instability. In macrophages, AMPK inhibits de novo cholesterol synthesis and promotes autophagic flux required for cholesterol efflux. At the same time, it limits the sustained activation of the NLRP3 inflammasome. Recent studies on the β1 subunit‐selective agonist PF‐06409577 in two atherosclerotic models have shown triple reductions in inflammatory transcript profiles, lipid synthesis, and lesion burden. This effect depends on myeloid cell‐derived AMPK β1 expression, providing direct evidence for “cell lineage‐specific” regulation [90, 91, 92, 93, 94].

Regarding vascular smooth muscle cells, the role of mTORC1 in phenotypic switching and migration has been supported by multiple studies. Through the regulation of the Raptor and TSC axes, AMPK inhibits excessive proliferation of smooth muscle cells and cytoskeletal remodeling. This delays medial thickening and lumen stenosis. Changes in shear stress and wall tension can also reduce the probability of EndMT and the transformation of adventitial fibroblasts to an inflammatory phenotype through AMPK‐mediated coordination of metabolism and stress kinases. Relevant evidence is continuously strengthened by metabolic studies and reviews on wall shear stress responses [6, 45, 52, 86, 95, 96].

The aspect of inflammation deserves to be discussed separately. In the cardiovascular system, NLRP3‐driven interleukin cascades and pyroptosis not only expand the necrotic core within plaques but also inhibit the stabilization of the fibrous cap. Multiple updated reviews and experimental studies have concluded that AMPK, as a metabolism–inflammation hub, can downregulate NLRP3 assembly and ASC recruitment. This reduces the maturation of IL‐1β and IL‐18. Integrating this axis with cholesterol metabolism, autophagic flux, and endothelial metabolic programming can construct a “metabolic anti‐inflammatory pathway” that runs through endothelial cells to immune cells. This pathway also occupies a central position in the cross‐disease network shown in Figure 4; see also references [76, 77, 97, 98, 99].

β‐Subunit selectivity and myeloid‐targeting strategies can reduce the risk of delayed healing caused by excessive inhibition of smooth muscle cell proliferation. They can also avoid the side effects of myocardial glycogen accumulation caused by long‐term systemic activation [90, 91, 92, 93, 94, 100]. In early plaques dominated by endothelial dysfunction and mild macrophage foam cell formation, endothelial metabolic correction and mild AMPK activation can be used as a “homeostasis repair” strategy. In vulnerable plaques with obvious necrotic cores and hemorrhage, priority should be given to inhibiting inflammasomes and correcting glucose‐lipid metabolic imbalance. The activation magnitude and duration of AMPK should be controlled below the threshold of “anti‐inflammation priority” [76, 77, 97, 98, 99]. The combination of AMPK modulators with mTORC1 inhibitors, PPAR pathway regulators, and SGLT2 inhibitors needs to be dynamically evaluated through imaging and inflammatory markers. This avoids adverse effects on the vascular repair stage [79, 80, 81, 82, 83, 86, 95, 96].

### 3.5 Other Cardiovascular Diseases

In specialized fields such as pulmonary circulation and cerebrovascular malformations, metabolism and mechanics also converge through AMPK. In pulmonary arterial hypertension models, PFKFB3‐driven enhancement of endothelial glycolysis and aerobic glycolysis in vascular myocytes jointly promote remodeling. The rebalancing of glycolysis and mitochondrial oxidation by AMPK can reduce the extent of abnormal neovascular sprouting and medial thickening. Recent studies have revealed that the stress hormone FGF21 can construct a two‐tier energy flux system by activating the LKB1–AMPK–mTOR pathway. It stimulates hepatocytes to produce ketone bodies such as β‐hydroxybutyrate as “super fuels” for the myocardium. At the same time, it regulates the metabolite decomposition and mitochondrial homeostasis in the heart. This protective mechanism was also observed in models of pulmonary arterial hypertension complicated with cardiac insufficiency [101]. Meanwhile, Notch‐related shear stress signals have been confirmed to be involved in the formation of arteriovenous malformations and endothelial‐to‐mesenchymal transition. The interaction between AMPK and Notch during mechanical adaptation deserves special attention. Recent studies based on human and animal data suggest that in the context of increased energy pressure and mechanical stress in malformed endothelial cells, decreased AMPK activity can change in the same direction as EndMT‐promoting factors. The combination of small‐molecule Notch signaling blockade and metabolic correction may become a pathway to reduce lesion expansion and leakage [52, 102, 103, 104, 105, 106, 107].

In valvular heart disease, osteoblast‐like transdifferentiation of aortic valve interstitial cells is the core driver of calcification progression. Recent studies starting from metabolism suggest that upregulation of RhoA and ROCK can drive “Warburg‐like” metabolic switching. This further increases RUNX2 expression and is accompanied by limited AMPK signaling. At the cellular and animal levels, AMPK activation and its downstream enhancement of antioxidant effects and autophagy can inhibit osteogenic programs. SGLT2 inhibitors, such as canagliflozin, delay the progression of valvular calcification through the crosstalk between the AMPK and Nrf2 pathways. AMPK can also regulate the ubiquitination modification of the autophagy‐related protein Beclin‐1 through phosphorylation. This accelerates RUNX2 degradation and inhibits the formation of calcified nodules, providing a new molecular target for metabolic regulation [108].

Central regulation of hypertension is another aspect worthy of attention. In the past 2 years, multiple studies have linked the upregulation of oxidative stress and inflammatory signals in the hypothalamic paraventricular nucleus to sympathetic excitation. In this context, micro‐activation of AMPK in the central nervous system has been shown to reduce reactive oxygen species, inhibit the NFκB pathway, and alleviate sympathetic outflow. This produces a mild “desensitization” effect between renal vasoconstriction and cardiac afterload. These findings extend the “metabolic sensing” concept of AMPK to autonomic nerve regulation. They also provide an experimental basis for the integrated management of refractory hypertension [109, 110, 111, 112, 113, 114]. The sodium handling pathway in peripheral renal tubules is mainly regulated by the WNK and SPAK pathways. Currently, evidence regarding the direct regulation of distal convoluted tubule NCC by AMPK remains inconsistent and should be stated with caution. There is relatively solid evidence that AMPK plays a role in protecting renal tubular epithelial cells against lipotoxicity and lysosomal stress. This suggests that in the context of the interplay between high salt, hypokalemia, and metabolic syndrome, AMPK may indirectly improve blood pressure variability and organ damage through renal protection. This requires verification in combination with renal imaging and urinary markers [115, 116].

The understanding of metabolism in congenital heart disease is being reshaped by myocardial organoids and transcriptomics. In the mature myocardium, the shift in energy preference from glucose to fatty acid oxidation is governed by networks involving AMPK and ERR. Recent organoid studies demonstrate that metabolic maturation strategies targeting these pathways can improve the synchronization of contraction and calcium handling in vitro. Notably, by targeting the FGF21–LKB1–AMPK pathway, exogenous FGF21 supplementation accelerates the metabolic maturation of myocardial organoids. This activation increases the expression of fatty acid oxidation‐related enzymes by more than 40% and significantly improves the synchronization of contractile force. These findings suggest that perioperative supplementation of FGF21 may become a novel method for myocardial protection [48].

Returning to the cross‐disease common network emphasized in Figure 4, the key links in the aforementioned different diseases can be categorized into three “shared pathways.” In the coupling of mechanics to metabolism, the axis from VE‐cadherin to LKB1 and AMPK is the master switch for endothelial homeostasis. In the coupling of metabolism to inflammation, the regulation of NLRP3 and autophagic flux by AMPK determines the “functional orientation” of immune cells in plaques, myocardium, and pulmonary circulation. In the coupling of metabolism to growth and matrix, the inhibition of mTORC1 and protein synthesis by AMPK, together with its promotion of autophagy, jointly define the speed and magnitude of hypertrophy, fibrosis, and calcification. Therapeutic strategies based on these three pathways are more likely to achieve “directional benefits” across different disease types. They also facilitate the evaluation of cross‐disease universal efficacy indicators using molecular imaging, metabolic profiling, and electrophysiological endpoints in clinical trial design.

## 4 Beyond the Heart: The Role of LKB1–AMPK in Systemic Diseases

Beyond the cardiovascular system, the LKB1–AMPK pathway also plays a key regulatory role in various systemic diseases. Under the long‐term influence of metabolic syndrome and type 2 diabetes, energy‐sensing imbalance and low‐grade inflammation reinforce each other, gradually reshaping the cardiovascular system. In neurodegenerative diseases, one of the earliest functional dysfunctions is also the disruption of energy supply–demand balance. In the field of oncology, the role of the LKB1–AMPK pathway exhibits a remarkable “duality,” with its functional orientation closely linked to the genetic background of tumor cells, the nutritional microenvironment, and redox status.

### 4.1 Metabolic Syndrome and Type 2 Diabetes: The Root of Cardiovascular Comorbidities

In adipose tissue, deletion of LKB1 in Group‑2 innate lymphoid cells disrupts mitochondrial homeostasis, elevates PD‑1, and dampens ILC2 responses, leading to insulin resistance; blocking PD‑1 or restoring mitochondrial balance partly reverses this phenotype [117]. In the atherosclerosis models, selective activation of macrophage AMPKβ1 with PF‑06409577 simultaneously reduces cholesterol synthesis and inflammatory transcriptional programs, lowering plaque burden in both ApoE^−/−^ and LDLR^−/−^ mice [90]. Upstream, tankyrase constrains LKB1 and AMPK, and thereby perturbs metabolic homeostasis, pointing to a pathological “brake” that may be druggable [118]. These pathways and candidate interventions are summarized in Table 1. A human multiorgan‑on‑chip study further indicates that adipose tissue inflammation is a dominant driver of hepatic and pancreatic insulin resistance, and pharmacological testing suggests that GLP‑1 receptor agonists primarily act at the adipocyte level [119]. Phosphoproteomic mapping reveals that insulin resistance entails network‑level rewiring, supporting multi‑node intervention at energy‑sensing hubs [120].

**TABLE 1:** Translational landscape and drug atlas of the LKB1–AMPK pathway: Mechanism‐aligned summary of preclinical and clinical evidence with representative modulators.

Mechanism	Modality	Representative agent(s)	Preclinical evidence (model → key finding)	Clinical evidence (phase/indication/outcome)	Development status (2025)	Reference
Direct AMPK activation (ADaM site), β1‐selective	Oral small molecule; AMPKβ1‐selective	PF‐06409577	Mouse and ApoE^−/−^ atherosclerosis models → ↓ plaque and inflam. transcriptome via myeloid AMPKβ1; rodent/primate liver → ↓ lipogenesis, ↓ cholesterol synthesis	No pivotal clinical outcomes; translational PD evidence published	Preclinical/Early translational	[19, 90]
Direct AMPK activation (ADaM site), pan‐activator	Oral small molecule; nonselective across β subunits	MK‐8722	Rodents/Primates → ↑ glucose disposal, ↓ glucose; chronic dosing → cardiac hypertrophy	Early human exposure; development curtailed due to preclinical cardiac hypertrophy signal	Discontinued/Paused	[27, 121]
Direct AMPK activation (ADaM site), clinical PoC	Oral small molecule; first‐in‐class direct activator	PXL770	NAFLD/NASH preclinical → ↓ steatosis, improved insulin sensitivity (reported)	Phase IIa (NAFLD/NASH) → ↓ liver fat, metabolic improvements vs. placebo	Completed Ph IIa; further development reported by sponsor	[28, 122]
Direct AMPK activation (pan)—“exercise‐mimetic” class	Oral pan‐AMPK activator	O304 (ATX‐304)	DIO mice and aged mice → ↑ microvascular perfusion, ↑ exercise capacity, ↓ insulin resistance; CV benefits in obesity models	Phase IIa PoC in T2D → ↓ fasting glucose, ↓ BP, ↑ microvascular perfusion	Translational/Early clinical; ongoing program updates	[84, 123]
Direct AMPK activation (ADaM site)—tool compound	Oral small molecule (tool); β1‐biased	A‐769662; 991 (tool)	Rodent hearts/immune cells → anti‐inflammatory and metabolic effects via AMPK	None (tools for mechanism)	Preclinical tools only	[124, 125]
Upstream LKB1–AXIN–lysosome scaffolding; Complex I–energy stress	Indirect AMPK activation via mitochondrial complex I inhibition and LKB1–AXIN assembly	Metformin	Cells/animals → LKB1–AXIN translocation to lysosome; AMPK activation; metabolic reprogramming	Extensive clinical use in T2D; pleiotropic CV risk signals (observational)	Marketed (indirect AMPK modulator)	[126, 127, 128, 129]
Indirect AMPK engagement; cardiometabolic remodeling	SGLT2 inhibition (multi‐modal; AMPK activation shown in preclinical cardio‐renal tissues)	Dapagliflozin; empagliflozin	Cardiomyocytes/rodents → ↑ AMPK activity; ↓ inflammation/oxidative stress; anti‐fibrotic signaling	Phase III HF programs (HFrEF/HFpEF) → ↓ CV death/HF hospitalization (DAPA‐HF, EMPEROR); broad HF benefit irrespective of T2D	Marketed for HF/CKD/T2D	[130, 131, 132]
Direct AMPK activation (salicylate site on β1)—legacy drug	Allosteric AMPK activation by salicylate binding at A‐769662 site	Salsalate/High‐dose aspirin (salicylate)	Hepatocytes/adipocytes and AMPK‐KO mice → direct AMPK activation; metabolic effects lost in AMPK‐KO	Older PoC in T2D (glycemic control signals); not widely adopted for AMPK targeting	Repurposing interest; limited by dose/tolerability	[125, 131]

Abbreviations: ADaM, allosteric drug and metabolite (AMPK β‐subunit allosteric pocket); HF, heart failure; HFrEF/HFpEF, HF with reduced/preserved ejection fraction; MASLD, metabolic dysfunction‐associated steatotic liver disease; NAFLD/NASH, non‐alcoholic fatty liver disease/steatohepatitis; PD, pharmacodynamics; PoC, proof‐of‐concept; RCT, randomized controlled trial.

Translational data align with these mechanisms. By leveraging energetic and osmotic effects along this axis, population trials have demonstrated benefit. In HF with reduced ejection fraction, dapagliflozin and empagliflozin each reduced the composite of cardiovascular death or heart‑failure hospitalisation, irrespective of diabetes status [131, 132]. In patients with preserved or mildly reduced ejection fraction, both agents decreased worsening‑HF events [133, 134], and consistency across the EF spectrum has been validated in independent cohort analyses [135]. In chronic kidney disease, the two agents slowed eGFR decline and reduced kidney outcomes [136, 137]. Subgroup analyses show that empagliflozin's efficacy is maintained across background diuretic exposure, though volume management requires clinical attention [138]. For weight‑centered strategies, once‑weekly semaglutide lowered major adverse cardiovascular events in predominantly nondiabetic patients with established atherosclerotic disease, and a prespecified analysis reported improvements among those with prior HF [139, 140]. These population findings match the network depicted in Figure 4: adipose–immune disequilibrium reshapes whole‑body insulin sensitivity through the hepato‑pancreatic axis, and ultimately influences myocardial energetics and volume load (see Figure 4).

With impaired kidney function, the glucose‑lowering component of SGLT2 inhibition diminishes, and rare euglycemic ketoacidosis can occur under perioperative or fasting stress [141]. GLP‑1 receptor agonists face gastrointestinal intolerance and adherence issues that may limit steady exposure in real‑world settings. Direct AMPK agonists have shown early‑phase signals of reduced hepatic de novo lipogenesis and improved insulin sensitivity, yet tissue‑selective delivery and sufficient target engagement—without a systemic “energy brake” adverse profile—remain key pharmacokinetic and formulation challenges [28, 142].

### 4.2 Neurological Disorders: Linking Brain Energy Crisis to Neurodegeneration

In neurodegeneration, one of the earliest failures is the balance between energy supply and demand. The LKB1–AMPK hub in neurons coordinates mitochondrial quality, proteostasis, and synaptic plasticity, with cell‑type and time‑window specificity. In Alzheimer's disease models, neuron‑specific repression of AMPKα1 alleviated hippocampal pathology and improved cognition, highlighting isoform selectivity as a potential entry point [143]. In primary neurons, the adiponectin receptor agonist AdipoRon rapidly activates AMPK, suppresses GSK3β, and promotes autophagic clearance of hyperphosphorylated tau; these effects disappear with pharmacological AMPK inhibition, demonstrating pathway dependence [144]. In vivo interventions include photobiomodulation, which increased AMPK activity and improved blood–brain barrier integrity and amyloid burden in APP/PS1 mice [145]. Crocetin enhanced autophagy and reduced Aβ through LKB1‑dependent AMPK activation [146]. More recent work shows that metabolic modulation outside of acarbose‑like agents can restore an impaired AMPK–ULK1 cascade in AD brain and rescue mitochondrial structure–function and neuroinflammation [147]. Notably, AMPK activation does not necessarily raise autophagy under basal neuronal conditions, underscoring the need to control amplitude and timing [148]. These observations align with the “energy–proteostasis” framework illustrated in Figure 4 and argue for interventions placed at the intersection of energy deficit and proteotoxic stress.

In Parkinson's disease, the initiation of mitophagy connects directly to this axis. Within minutes of mitochondrial stress, AMPK primes selective autophagy via ULK1‑dependent phosphorylation of Parkin at Ser108 [149]. Across models, moderate AMPK activation attenuates α‑synuclein aggregation and dopaminergic neuron loss [150], whereas conditional deletion of AMPK in dopaminergic neurons accelerates degeneration [151]. Conversely, under α‑synuclein fibril‐induced pathology, excessive AMPK activation can impair kinesin‑mediated axonal transport; partial pharmacological inhibition reverses this phenotype, emphasizing dose‑and‑window effects [152]. Beyond PD, AMPKα2 activation is protective in chronic cerebral hypoperfusion [153], while in some early phases of focal ischemia, AMPK inhibition reduces infarct size [154]. In migraine models, AMPK activation shifts microglia toward a reparative phenotype and dampens central sensitization [155]. Translationally, a 3‑month randomized, double‑blind trial in PD did not show metformin‑induced improvement in UPDRS, with signals limited to inflammatory and neurotrophic markers, suggesting constraints in sample size and exposure [156]. Human genetics in stroke supports an association between genetically proxied, metformin‑mediated AMPK activation and improved functional outcome [157]. In line with the “neurology” entries of Table 1, we favor two routes: selective control of AMPK isoforms/complexes with precise dosing windows, and gentle upstream activation via metabolic receptors to balance brain exposure and safety.

### 4.3 Cancer: The Dual Role of AMPK in Tumor Suppression and Metabolism

**FIGURE 6:**
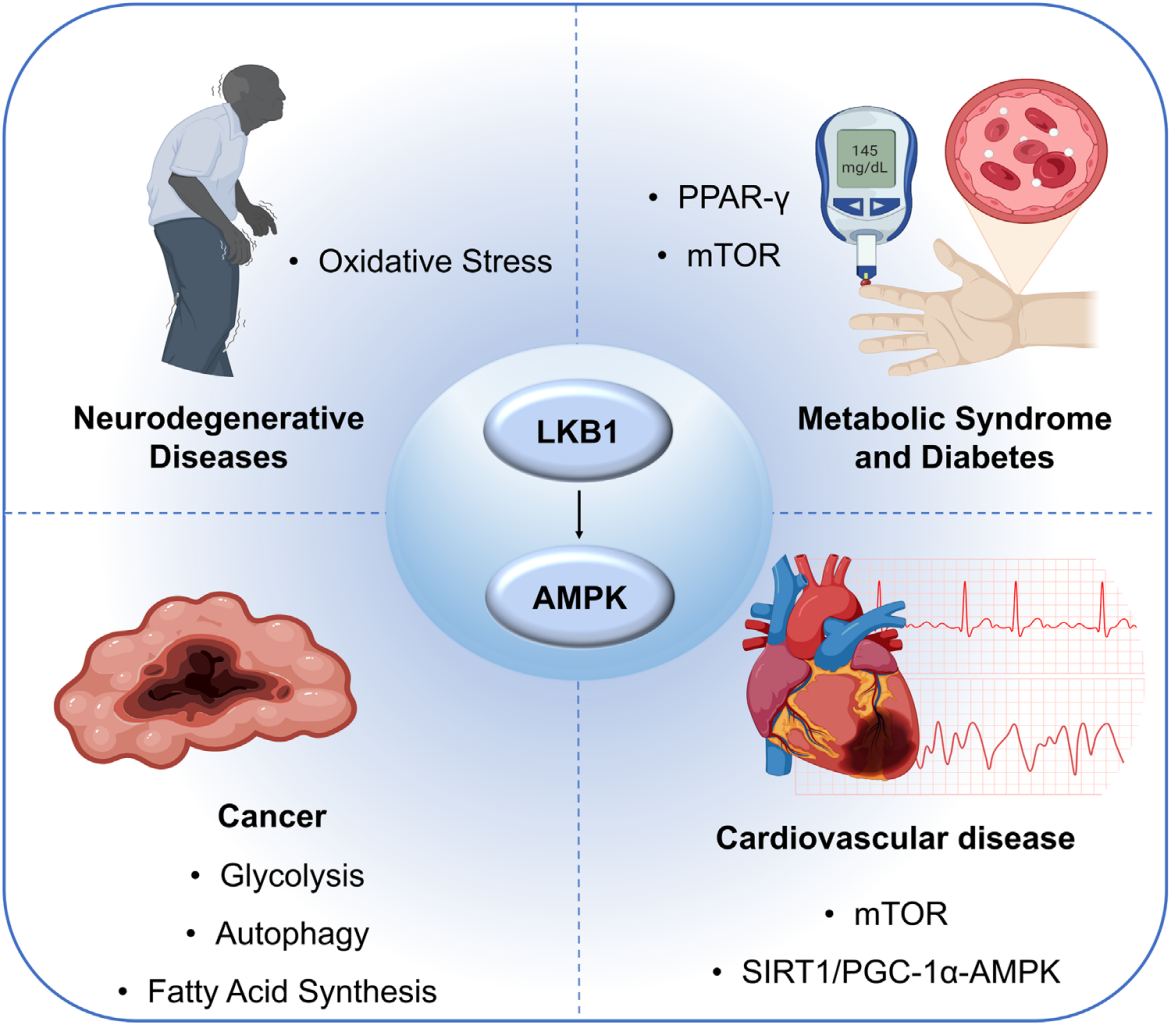
The central regulatory role of the LKB1–AMPK pathway in various diseases. Dysfunction of this pathway can lead to pathological processes in cardiovascular diseases (atrial fibrillation, myocardial infarction, myocardial hypertrophy, atherosclerosis), metabolic diseases (diabetes and kidney disease), neurodegenerative diseases (Alzheimer's disease, Parkinson's disease), and tumors (LKB1 mutation‐driven metabolic reprogramming). Targeting this pathway offers new therapeutic strategies across these diseases. Some elements in this figure were created using BioRender.

In solid tumors, loss of LKB1 and dampened AMPK signaling often track with aggressive features, yet AMPK can either suppress cancer or help it survive metabolic stress. On the suppressive side, in KRAS‑driven lung cancer, blocking autophagy together with MEK inhibition induces ferroptosis on the Lkb1‑deficient background, indicating that when LKB1 is absent, and redox buffering is thin, cutting off nutrient recycling can push tumors into irreversible damage [158]. Within the same lineage, ablation of G6PD markedly suppresses KRAS/LKB1‑mutant lung cancer but has little effect on KRAS/TP53 tumors, showing that LKB1 loss creates a dependency on cytosolic NADPH and de novo lipogenesis—an actionable metabolic liability [159]. Under energy stress, AMPK directly phosphorylates PD‑L1 and promotes its endocytosis and degradation, potentially relieving immune suppression [160]. In LKB1‑mutant settings with impaired antigen presentation and innate responses, ULK1 inhibition restores antigen presentation and T‑cell infiltration, pointing to a tractable node downstream of AMPK to “warm up” cold tumors [161]. These mechanisms are integrated in Figure 6 into a “metabolic vulnerability” track and an “immune‑modulation” track and crossreferenced to Table 1.

Contradictory evidence should not be minimized. AMPK can also sustain survival under matrix detachment and nutrient scarcity by maintaining NADPH and mitochondrial quality, thereby promoting anchorage‑independent growth and pre‑metastatic adaptation [162]. In the immune microenvironment, the ACC–AMPK pathway can support effector T cells in some contexts but stabilize regulatory T cells and reinforce immunosuppression in others, depending on cell type and nutrient availability [163, 164, 165]. Thus, indiscriminate, global AMPK activation may benefit tumors in certain scenarios. Consistent with this, complex‑I inhibition can sensitize LKB1‑deficient cancer cells to GPX4 blockade and ferroptosis, and in the LKB1‑loss background, combining KRAS‑pathway inhibition with blockade of autophagy initiation may be a more coherent strategy than blunt AMPK activation [158, 166].

Clinical translation reflects such a complicated situation. Metabolic‑constraint combinations are mechanistically aligned to LKB1 loss: complex‑I inhibition increases susceptibility to GPX4 inhibitors in LKB1‑deficient models [166], and early studies of KRAS‑pathway blockade plus autophagy inhibition have produced objective responses or subgroup signals [158]. However, the tolerability limits the application. The oral OXPHOS inhibitor IM156 established a recommended dose in first‑in‑human testing, but nausea and fatigue restricted upward titration [167]. Phenformin combined with BRAF/MEK achieved responses in early testing but caused reversible lactic acidosis, mandating tight metabolic monitoring [168]. In a greater than 3000‑patient Phase III breast cancer study, add‑on metformin did not improve invasive disease‑free survival, suggesting that single‑agent metabolic reprogramming is insufficient to change outcomes [169]. Pharmacokinetic studies show orders‑of‑magnitude variability in intratumoral metformin concentrations across patients, influenced by transporter expression and tissue perfusion, which likely contribute to inconsistent clinical signals [170]. Given these realities, Table 1 organizes LKB1–AMPK‑relevant agents by target node and clinical readiness, and annotates dosing window, dose‑limiting toxicities, and candidate populations for alignment with the narrative above.

The balance of AMPK's “two faces” depends on oncogenic context, nutrient and redox pressure, and the metabolic state of immune cells. Rather than a binary “activate versus inhibit” logic, it is advocated that phase‑of‑disease and niche‑aware designs: in LKB1‑deficient, high‑NADPH, and lipogenesis‑dependent tumors, prioritise complex‑I inhibition plus ferroptosis induction with autophagy‑initiation blockade; in immunosuppressed tumors, combine strategies that lower PD‑L1 stability or restore antigen presentation with checkpoint blockade [161, 171]. This modular, defect‑anchored approach better fits the current evidence and is more likely to translate under real‑world pharmacokinetic and tolerability constraints.

In summary, three cross‐disease common patterns emerge in the LKB1–AMPK pathway. First is the coupling of mechanical and metabolic signals, which influences the homeostasis of endothelium and smooth muscles. Second is the mutual regulation between metabolism and inflammation, involving cholesterol synthesis pathways, autophagy flux, and inflammasome gating. Third, the equilibrium between metabolism and growth/matrix remodeling involves coordinated inhibition of protein synthesis and mitochondrial renewal. These patterns provide a clearly structured starting point for developing therapeutic strategies.

## 5 Therapeutic Targeting of the LKB1–AMPK Pathway: Opportunities and Challenges

The value of the LKB1–AMPK pathway does not lie in indiscriminately “turning AMPK on,” but in precisely tuning disease stage, tissue context, and subcellular microdomains so that the beneficial effects of energy homeostasis are amplified at the right place and time while minimizing compensatory and off‐target risks. Evidence from structure–activity exploration of direct agonists and from subcellular imaging biosensors is making this three‐dimensional “microdomain–temporal–intensity” strategy concrete, providing clearer design coordinates for drugs [29, 38, 41, 172].

There are direct and indirect strategies regarding therapeutic targeting of the LKB1–AMPK pathway.

### 5.1 Direct Activation Strategies: Isoform Selectivity and Exposure Control

Direct activation strategies are shifting from broad activation to structure–activity‐driven and isoform‐selective approaches. The oral direct agonist PXL770 produced improvements in hepatic steatosis and metabolic indices in a Phase IIa NAFLD cohort, supporting the human feasibility of “AMPK as a drug target” [28]. PF‐06409577, which preferentially targets AMPKβ1‐containing complexes, suppressed macrophage inflammation and cholesterol synthesis, and reduced plaque burden in murine atherosclerosis, suggesting that lineage‐directed selectivity may increase efficacy while widening the safety margin [90]. At the same time, spatial compartmentalization at the subcellular level and temporal switching of autophagy programs reveal that excessive or prolonged activation can provoke countervailing effects, indicating that exposure profiles and tissue distribution should align with microdomain biology [29, 38, 41].

### 5.2 Indirect Modulation Strategy: Physiological Amplification and Mechanochemical Coupling

Indirect modulation offers a “physiologic amplification” route. Mechanical forces on endothelial adhesion molecules can activate AMPK through LKB1, enhancing glucose uptake and eNOS activity and promoting vasodilation, implying that the force–adhesion–metabolism axis could be amplified pharmacologically or by devices for minimally invasive translation [45]. Among natural small molecules, the rare ginsenoside Rh4 has been identified as a novel AMPK agonist that ameliorates high‐fat diet‐induced endothelial dysfunction while boosting mitochondrial biogenesis and nitric oxide production, pointing to a gentle, long‐term corrective strategy for disordered metabolism [47].

### 5.3 Targeting and Translation: Inhibition or Correction and Translational Medicine Considerations

In disease‐specific settings, “inhibition or correction” can be equally critical. The Bruton's tyrosine kinase inhibitor ibrutinib can provoke arrhythmias, in part through impaired myocardial AMPK activity, and pharmacologic enhancement of AMPK shows protective potential [58]. In animal models, metformin mitigated ibrutinib‐associated ventricular arrhythmias and myocardial dysfunction by augmenting AMPK and PI3K–AKT signaling, suggesting that a metabolic “protective kit” could be integrated into onco‐cardiology management [61]. With respect to atrial vulnerability, exogenous succinate lowers AMPK phosphorylation via SUCNR1, impairs mitochondria, and drives atrial fibrosis, whereas AMPK activators reverse this phenotype, revealing druggable nodes at the metabolism–inflammation interface [50].

At the systems level, pharmacokinetic constraints and indication boundaries must be anticipated. AMPK agonists may trigger endocrine pathways that suppress appetite and induce weight loss; evidence from rodents and humans indicates that metformin regulates bodyweight through a kidney–GDF15–area postrema axis, which raises stricter requirements for dosing and monitoring in patients at risk of malnutrition or cachexia [173].

In oncology, simply dialing up AMPK does not guarantee net benefit. LKB1‐deficient, KRAS‐driven lung cancers rely on autophagy to sustain metabolic plasticity, and autophagy inhibition shows synthetic‐lethal activity in this background, supporting “genotype‐stratified reverse” therapeutic strategies [174]. Targeting mitochondrial complex I can heighten ferroptosis sensitivity, exposing a metabolic vulnerability in LKB1‐inactivated tumors, while inhibiting the spindle assembly checkpoint kinase MPS1 can increase immunogenicity and create combination opportunities with immune checkpoint blockade in KRAS/LKB1 co‐mutant lung cancer [166, 175].

Looking forward to the future, development resembles deploying across the axes of disease stage, tissue, and microdomain: harnessing the assembly and partitioning of LKB1–AMPK at the Golgi, lysosomes, and mitochondria, and using localization and anchoring determinants to achieve microdomain‐targeted medicinal chemistry and delivery. Coupling‐stratified follow‐up, biomarker gating, and dosing designs that favor short exposure with rapid clearance can raise the probability of reproducible net clinical benefit [176]. To connect with the next section on conclusions and outlook, the core message here is to prioritize stage‐wise and microdomain specificity, integrating direct and indirect activation, corrective inhibition, and immuno‐metabolic combinations, thereby moving from mechanistic insight toward precise therapies that are replicable, monitorable, and stratified.

## 6 Conclusions and Future Perspectives

The LKB1–AMPK pathway stands as a cornerstone of cellular metabolism, whose integrity is fundamental to health and whose dysregulation is a common thread in a strikingly diverse array of human diseases. This review has consolidated evidence establishing its central role in cardiovascular pathologies—from arrhythmias to HF and atherosclerosis—where it acts as a critical nexus linking energy deficit to functional decline. Furthermore, its influence extends systemically, underpinning the metabolic derangements in diabetes, contributing to the energy crisis in neurodegenerative disorders, and serving as a potent tumor suppressor in oncology. This pleiotropy underscores the LKB1–AMPK axis not merely as a pathway but as an integrative network whose modulation holds immense therapeutic promise.

The burgeoning interest in targeting this pathway is validated by the long‐standing use of metformin and the development of novel, direct AMPK activators. However, the journey from mechanistic understanding to clinical application is fraught with challenges. The dual role of AMPK in certain cancers, its complex interplay with pro‐tumorigenic signals, and the critical issue of tissue‐specific outcomes highlight the limitations of a one‐size‐fits‐all approach. The very universality of the pathway is its greatest therapeutic hurdle, as evidenced by the risks of off‐target effects and the variable responses influenced by genetic background and disease state.

Looking forward, several key directions will define the next chapter of LKB1–AMPK research and its translation into medicine:

- Achieving Tissue and Context Specificity: The paramount challenge is the development of strategies to modulate the pathway in a cell‐type and disease‐specific manner. Future efforts should focus on designing allosteric modulators that target specific AMPK isoforms or complexes, exploiting tissue‐specific delivery systems (e.g., nanoparticle‐based carriers or viral vectors), and identifying downstream effectors that mediate the pathway's beneficial effects, in particular, organs without systemic consequences.
- Integration of Multi‐Omic Data for Biomarker Discovery: The pathway's central role makes it a rich source for biomarker development. Future studies should integrate genomics, transcriptomics, and proteomics from large patient cohorts to define “LKB1–AMPK signatures” that can predict disease susceptibility, progression, and therapeutic response. This will be crucial for enrolling the right patients in clinical trials and for the eventual realization of precision medicine.
- Exploring Novel Modalities Beyond Small Molecules: While pharmacological activation remains a primary goal, gene therapy approaches aimed at restoring LKB1 function in specific tissues (e.g., the heart or brain) represent a promising frontier. Similarly, the potential of perioperative supplementation of pathway agonists, such as FGF21, for myocardial protection merits rigorous clinical investigation as an acute intervention strategy.
- Decoding the Pathological Versus Physiological Switch: A deeper understanding of how LKB1–AMPK signaling is rewired in disease states is needed. Why does pathway activity become impaired in chronic conditions like HF or neurodegeneration despite the apparent need for its energy‐restoring functions? Elucidating the mechanisms of this dysregulation—whether through post‐translational modifications, altered subcellular localization, or interactions with disease‐specific proteins—will reveal new, more nuanced therapeutic nodes.

In conclusion, the LKB1–AMPK pathway is a gateway to understanding the metabolic basis of a vast disease spectrum. Its future as a therapeutic target lies not in broad activation, but in smart, context‐dependent manipulation. By leveraging insights from structural biology, advanced drug delivery, and human genetics, we can aspire to precisely tune this master regulatory system, turning its profound biological power into targeted therapies for some of the most complex and prevalent diseases.

## Author Contributions

**Zhuo Chen** and **Guo‐Wei He** researched data for the article, discussed its content, and wrote the manuscript. **Qin Yang** discussed its content. All the authors reviewed/edited the manuscript before submission, and have read and approved the final manuscript.

## Funding

The authors are supported by the National Natural Science Foundation of China (82370350 and 82170353), Tianjin Key Medical Discipline Construction Project (TJYXZDXK‐3‐036C), and Special Fund for High Quality Development Project.

## Ethics Statement

The authors have nothing to report.

## Conflicts of Interest

The authors declare no conflicts of interest.

## Data Availability

The authors have nothing to report.
